# An Overlooked Entity in Diagnosing Hip Pain with Neuropathy in an Athletic Young Adult: A Case Report

**DOI:** 10.5704/MOJ.2211.021

**Published:** 2022-11

**Authors:** AF Mohd-Hanaffi, A Puji

**Affiliations:** 1 Sports Medicine Unit, Hospital Kuala Lumpur, Kuala Lumpur, Malaysia; 2 Department of Orthopaedics, Hospital Kuala Lumpur, Kuala Lumpur, Malaysia

**Keywords:** hip pain, osteoid osteoma, magnetic resonance arthrography (MRA)

## Abstract

Hip pain is frequently encountered in the athletic community. Femoro-acetabular impingement (FAI) is a common cause of hip pain in young adults. However, it is important to appreciate the uncommon diagnosis and the role of imaging for unexplained hip pain. The pathology behind a posterior hip pain is often misdiagnosed as the cause of hip pain is non-specific, extensive and elusive. We managed to detect the pathology through magnetic resonance arthrography (MRA) of hip with gadolinium enhancement after a series of inconclusive history, physical examination and imaging findings were completed. This particular case vignettes an overlooked osteoid osteoma that leads to the delay in diagnosis and increase morbidity.

## Introduction

Osteoid osteoma is a rare small benign osteogenic tumour which is usually found in young patient1. It accounts for approximately 14% of all benign bone tumours where it mostly seen in a long bone particularly at the proximal femur^1,2^. Though rare, they are the indicators of increased morbidity due to the misdiagnosis. An accurate history, clinical examination and adequate imaging are needed to diagnose osteoid osteoma.

We report a case of a 20-year-old young, fit and active male, who is presented with a complaint of long-standing unexplained hip pain with meralgia paresthetica and was diagnosed with osteoid osteoma at the proximal shaft of right femur that only detected through magnetic resonance arthrography (MRA) of hip with gadolinium enhancement.

## Case Reports

A 20-year-old patient, actively involved in parkour presented with progressive dull right hip pain radiating to the anterolateral aspect of the leg caused by osteoid osteoma of proximal shaft of femur is described. He was managed as herniated disc for eight months by primary care before the accurate diagnosis was established in sports clinic. He had no history of trauma and constitutional symptoms. The pain worsened upon walking, standing, prolonged sitting and it was partially alleviated by analgesic. As the pain progressed, he had difficulty in walking, affected his daily activities and limited his participation in any sports activities. On examination, he was healthy and fit looking. He had normal posture and antalgic gait.

He is 163cm height and weight 50kg. The vital signs were within normal limits. The chest was clear to auscultation bilaterally with no chest wall abnormalities. The abdomen was soft, non-tender, and non-distended. Inspection of the spine and lower extremities revealed no erythema, effusions, or signs of trauma. The only positive findings revealed a vague discomfort around right hip area and right quadriceps muscular atrophy with the circumference difference of 3cm compared to the left side. Sensation was impaired at L2, L3 and L4. Other examinations and special tests were unremarkable. At that time all the conventional radiographs were reported as normal. The working diagnoses also included femoro-acetabular impingement, acetabular tear, paralabral cyst, piriformis syndrome and herniated disc.

**Fig. 1.**
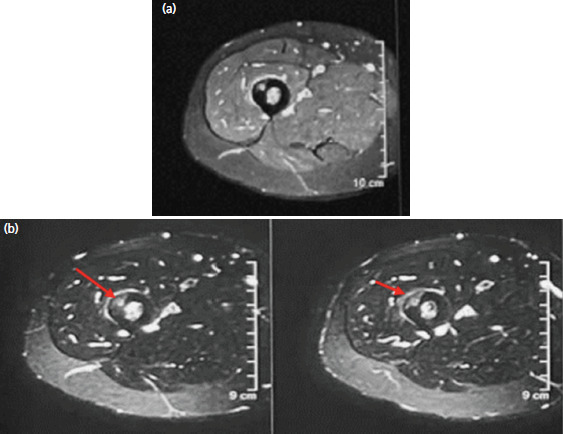
(a) A well-defined T1W hyperintense intracortical lesion at anterolateral aspect of proximal shaft of femur measures 0.5x0.7cm. (b) The lesion exhibits an abnormal signal on T2W sequence with minimal surrounding oedema involving the cortex and minimal extension involving marrow. The lesion demonstrates enhancement on post gadolinium sequence.

An urgent MRA pelvic and hip with gadolinium contrast done showed a well-defined intracortical lesion at anterolateral aspect of proximal femur with minimal surrounding edema and no cortical break (Fig. 1a). In addition, there was gadolinium enhancement of the lesion in favour of osteoid osteoma (Fig. 1b). He was referred to the tumours management team for further evaluation and management

## Discussion

Osteoid osteoma is a rare small benign osteogenic tumour which is usually found in young patients ranging from 5 to 20 years old^1^. It accounts for approximately 14% of all benign bone tumours where it mostly seen in a long bone with at least 25% located at the proximal femur^1,2^. The typical clinical picture is that either the patient will experience nocturnal pain or may manifest various degrees of limping which is responsive to NSAIDS^1^. An early diagnosis is difficult to make due to perplexing and nebulous clinical and radiological findings. The lag in diagnosis has been reported up to 8.5 months^3^. Femoro-acetabular impingement and labral tear are the commoner diagnoses in young adults with hip pain, it accounts for 46% of hip pain presentations^4^. Repetitive micro-trauma is the main causative factor particularly when it involves jump-landing tasks which may consist of extreme range of motion. The manifestation can behave atypically like no night pain, less responsive to NSAIDS, can also be overlapping with referred pain from the lumbar spine and presence of lower limb neuropathy with an absent of radiolucent nidus surrounded by a variable area of reactive sclerosis. These can lead to a diagnostic challenge.

It is important to be familiar with the radiological findings in osteoid osteoma and its mimics. Classically, 42% of patients had radiolucent nidus and about 58% had osseous sclerosis and cortical thickening^4^. With a greater ability to visualise soft tissue and cartilage, MRI has become a vital imaging for evaluation of hip disorder. However, in the case of osteoid osteoma, MRI alone only contributes about 3% in making a correct diagnosis^4^. This is because the presence of localised cortical thickening and marrow edema can lead to erroneous alternative diagnoses. Majority of studies used the CT scan which is thought to be more sensitive to identify the nidus and to think of the possibility of an osteoid osteoma. However, recent studies showed gadolinium contrast MR demonstrated better sensitivity than CT scan^5^.

As increasing numbers of young, active patients are being evaluated for various causes of hip pain, clinicians should have a pensive index of suspicion for osteoid osteoma. Osteoid osteoma of the proximal shaft of femur exhibit progressive course and mimics a lumbar spinal root nerve compression which difficult to diagnose at early stage. Atraumatic young and active patient with history of chronic pain should be evaluated and be advised to undergo regular re-examination. Multiple CT scan should be considered in order to detect nidus. Thereby, it helps in early diagnosis and intervention. Therefore, it significantly prevents the delay in making an accurate diagnosis and treatment. Thus, it will improve the patient quality of life.
